# Mess. Scott & Taynton, on Cold Applications in Gout

**Published:** 1803-06-01

**Authors:** 


### *To the Editors of the Medical and Physical Journal.*

GENTLEMEN,

IN consequence of Dr. Kinglake's pressing invitation to medical men to communicate their experience in the use of cold applications in the cure of Gout and Rheumatism, Mr. Scott and Mr. Taynton, surgeons at Bromley, in Kent, cannot any longer defer making known, through the medium of your excellent publication, the result of their own practice in these disorders. Amongst several cases that have fallen under their care, they think the four following the most deserving of notice.

CASE I. On the 24th of February, 1803, they were sent for to a young married woman who had been ill three days. She was unable to stir hand or foot; her pulse was quick and hard; she had great thirst, and complained of severe pain in her feet and ancles, attended with swelling and inflammation; the wrists and elbows were also affected. Cloths wetted in a solution of muriated ammonia, were ordered to be constantly applied to the parts; and one grain of opium was given her at night. On the 25th they were agreeably surprized to find the swelling and inflammation nearly gone, and the pain much abated. On the 26th she was able to walk down stairs, and by the beginning of March was cured.

CASE II. On the 14th of April they visited a young man labouring under acute Rheumatism. The febrile symptoms were pretty severe; he was unable to walk; felt great pain in the ancles and knees, and in one elbow. As he was costive, he was ordered to take an opening medicine, and to apply constantly the cold solution. He soon experienced experienced ease; and though the pains shifted from one joint to another, and the weakness did not leave him so soon as in the former case, he was able to move about with the assistance of crutches in three days, and by the 28th was well.

CASE III. A gentleman's butler was attacked by Gout in the great toe of his left foot on the 11th of April in the morning. He had repeated exacerbations and remissions of pain till the 14th, when they saw him: they found the toe greatly inflamed, the skin shining, the parts exquisitely tender, and the patient not able to set his foot to the ground. It was wrapped up in flannel, which was immediately removed, and in its stead it was surrounded with wet cloths dipped in the cold solution, and an opening draught was given him. He found relief in half an hour, and on the following day could set his foot on the ground. On the 20th he was quite well.

CASE IV. A lady, upwards of sixty, habitually subject to Asthma and tedious fits of the Gout, whilst labouring under severe dyspnœa and cough, attended with pyrexia, was attacked with pain and inflammation in the instep and joint of the great toe. The parts were of *course* immediately envelloped by her attendants in a quantity of flannel, with a view, *no doubt,* of enticing all her other *complaints on this one spot.* The cough was treated as if she had had no gout; and the gout as if she had had no cough. The cold application was of great use; no unpleasant symptom occurred, and she was soon cured.

Mr. Scott and Mr. Taynton take this opportunity of returning their acknowledgments to Dr. Kinglake, for his valuable communication on this subject. They have every reason to think that his plan of treatment will prove highly beneficial to mankind; and, as they are in very extensive practice, they will neglect no opportunity of giving it a fair trial, and of making known the result of their failure as well as of their success.

*Bromley, Kent, May* 12, 1803.

